# Retraction: Modulation of miRNA-10a-mediated TGF-β1/Smads signaling affects atrial fibrillation-induced cardiac fibrosis and cardiac fibroblast proliferation

**DOI:** 10.1042/BSR-2018-1931_RET

**Published:** 2023-07-19

**Keywords:** Atrial fibrillation, Cardiac fibrosis, MiR-10a, Proliferation, TGF-β1/Smads

This article is being retracted from *Bioscience Reports* at the request of the Editorial Board following receipt of a notification from a reader, alerting the Editorial Office to images in in Figure 3A, which overlap with those also published in Cheng et al 2021 (doi: 10.1007/s12012-021-09661-2) and Ni et al 2017 (doi: 10.1155/2017/7083016). Readers have also identified that Western blots in Figures 6A and 7B appear highly similar to those published in Yang et al 2018 (doi: 10.1186/s12871-018-0607-4), Feng et al 2019 (doi: 10.1080/13880209.2019.1657153).

The authors have been contacted with regards to the retraction and have not responded to the Journal's queries or the concerns raised. Given the extent of the issues raised, the Editorial Board stand by the decision to retract the article.

